# The Relationship Between Obesity and Intestinal Alkaline Phosphatase: A Clinical and Biochemical Study

**DOI:** 10.5152/tjg.2026.26008

**Published:** 2026-06-05

**Authors:** Alper Uysal, Nilay Danış, Ali Şenkaya, Seymur Aslanov, Ilgın Yıldız Şimşir, Güliz Armağan, Rukiye Vardar

**Affiliations:** 1 Department of Gastroenterology, Kanuni Sultan Süleyman Training and Research Hospital, İstanbul, Türkiye; 2 Department of Gastroenterology, Dokuz Eylül University Faculty of Medicine, İzmir, Türkiye; 3 Department of Gastroenterology, Ege University Faculty of Medicine, İzmir, Türkiye; 4 Gastroenterology Outpatient Clinic, İzmir Medicalpoint Hospital, İzmir, Türkiye; 5 Department of Endocrinology, Ege University Faculty of Medicine, İzmir, Türkiye; 6 Department of Biochemistry, Ege University Faculty of Pharmacy, İzmir, Türkiye

**Keywords:** Endotoxemia, inflammation, intestinal alkaline phosphatase, intestinal barrier function, metabolic syndrome, obesity

## Abstract

**Background/Aims:**

Intestinal alkaline phosphatase (IAP) is a key regulator of gut barrier function, inflammation, and metabolic homeostasis. Human studies evaluating tissue IAP (tIAP) and serum IAP (sIAP) levels in obesity are limited. The current study aimed to investigate the association of sIAP and duodenal IAP levels with obesity and metabolic syndrome (MeS).

**Materials and Methods:**

Fifty-two adults undergoing diagnostic upper endoscopy were prospectively enrolled. sIAP and tIAP levels were measured using Enzyme-Linked Immunosorbent Assay (ELISA), and intestinal IAP expression was assessed by Western blotting. Participants were classified according to body mass index (BMI) and MeS status.

**Results:**

Both sIAP and tIAP levels showed strong inverse correlations with BMI (*P* < .01) and waist circumference. Obese individuals exhibited significantly lower IAP levels than normal-weight participants (*P* < .001). tIAP levels were significantly lower in subjects with MeS. Western blot analysis confirmed reduced IAP expression in obese individuals (*P* = .0139).

**Conclusion:**

Reduced systemic and local IAP levels are associated with obesity and MeS, suggesting that IAP may serve as a potential biomarker for metabolic disease.

**Main Points**

- Intestinal alkaline phosphatase (IAP) levels differ according to obesity status.
- Reduced IAP levels are associated with metabolic and inflammatory parameters in obese individuals.
- IAP may serve as a clinically relevant biomarker of obesity-related metabolic dysfunction.

## Introduction

Obesity is a multifactorial metabolic disorder characterized by abnormal adipose tissue accumulation and persistent low-grade systemic inflammation. This condition is closely associated with the development of insulin resistance, alterations in lipid metabolism, and an increased risk of cardiovascular disease. Beyond caloric imbalance, recent research has emphasized the pivotal role of the gut–liver axis and intestinal microbiota in the development of obesity and metabolic syndrome (MeS). Alkaline phosphatase (ALP), first described in 1907 by Suzuki et al,^1^ is a membrane-bound glycoprotein that, in humans, is classified into 4 isoenzymes according to tissue distribution: placental ALP, germ cell ALP, tissue-nonspecific ALP (expressed in the liver, bone, and kidney), and intestinal alkaline phosphatase (IAP).^2^ IAP is an endogenous brush border enzyme expressed in the entire intestinal mucosa, with highest expression in the duodenal mucosa. IAP is localized to the apical microvilli on the brush edge of enterocytes and is released into both the intestinal lumen and the bloodstream.^3^ IAP exerts several critical physiological functions, including detoxification of lipopolysaccharides (LPS), regulation of gut permeability, modulation of lipid absorption, and maintenance of microbiota homeostasis.^4^ With these functions, IAP plays an important role in chronic intestinal inflammation and energy balance. Animal studies have shown that IAP deficiency in mice leads to endotoxemia, insulin resistance, and obesity.^5^^,^^6^ Another animal study demonstrated a clear association between IAP and MeS, showing that IAP inhibits endotoxin absorption and both prevents and reverses high-fat diet–induced MeS.^7^ An early human study evaluating both duodenal tissue messenger ribonucleic acid (mRNA) and protein expression demonstrated that IAP levels were significantly decreased in newly diagnosed celiac disease, likely reflecting impaired intestinal barrier integrity given the role of IAP in lipopolysaccharide detoxification and innate immune regulation, with partial normalization following a gluten-free diet.^8^ Another human study by Malo et al^9^ demonstrated that IAP plays a protective role against type 2 diabetes mellitus (T2DM). The authors showed that fecal IAP levels were significantly lower in patients with T2DM compared to healthy controls and that decreased IAP levels were independently associated with an increased risk of diabetes. Human studies, however, remain limited and partially conflicting. Reduced fecal or serum IAP (sIAP) levels have been associated with obesity and MeS, but data on duodenal tIAP expression remain scarce.^10^ Given that IAP is secreted into both the intestinal luminal and the systemic circulation, simultaneous evaluation of serum and tissue levels may provide more reliable insights into its metabolic relevance.

The current study was designed to examine the association of obesity with both sIAP and duodenal IAP levels and to assess their relationship with MeS. It was hypothesized that obese individuals and those with MeS would exhibit lower IAP activity than normal-weight subjects.

## Materials and Methods

**Figure 1.**
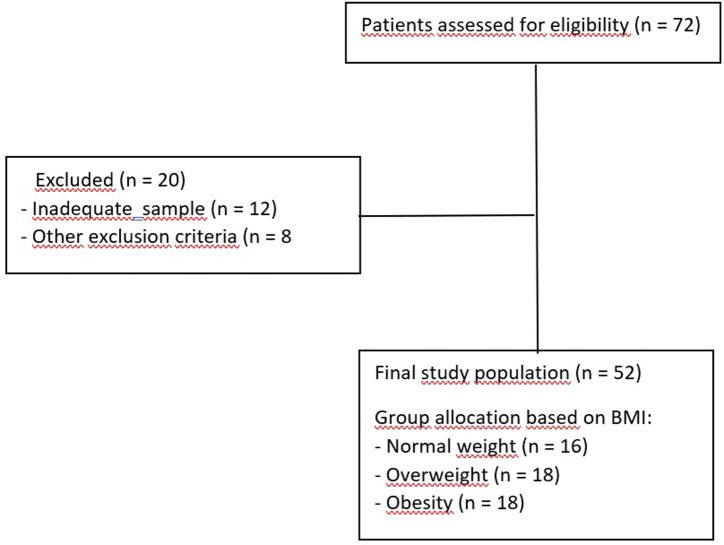
Patient selection flow diagram.

This prospective observational study included 52 adult patients scheduled to undergo upper gastrointestinal endoscopy in the Ege University Endoscopy Unit based on indications determined by a gastroenterologist. Eligible patients had serum ALP, fasting blood glucose, and lipid profile measurements available within at least 1 month prior to the procedure. Patients were enrolled between December 2017 and May 2018 (Figure 1). Exclusion criteria included chronic liver disease, renal dysfunction, inflammatory bowel disease, recent infection, malignancy, and antibiotic or probiotic use within the previous 3 months. Anthropometric measurements were obtained with participants wearing light clothing and no shoes. Body mass index (BMI) was calculated as body weight in kilograms divided by height in meters squared. Waist circumference was measured at the midpoint between the inferior border of the last rib and the iliac crest. After a 10-minute resting period, blood pressure was measured using an automated sphygmomanometer. Fasting venous blood samples were collected following an overnight fast of at least 12 hours for sIAP measurement.

Serum samples were centrifuged at 1000 x g for 10 minutes and stored at −80°C until analysis. Duodenal mucosal biopsy specimens were obtained from the second portion of the duodenum during endoscopy and immediately frozen in liquid nitrogen. IAP levels were quantified in both serum and tissue homogenates using a commercial ELISA kit (FineTest Human IAP ELISA Kit, Wuhan, China) according to the manufacturer’s instructions. Protein concentration was measured using the Lowry method, and IAP levels were expressed as ng/mL.

Participants were categorized into 3 groups based on BMI: normal-weight individuals with a BMI <25 kg/m^2^ (n = 16), overweight individuals with a BMI between 25 and 30 kg/m^2^ (n = 18), and obese individuals with a BMI >30 kg/m^2^ (n = 18). MeS was diagnosed in accordance with the criteria established by the National Cholesterol Education Program Adult Treatment Panel III. Individuals fulfilling at least 3 of the following 5 criteria were considered to have MeS: waist circumference ≥102 cm in men or ≥88 cm in women; fasting plasma glucose ≥110 mg/dL or current use of antidiabetic therapy; triglyceride levels ≥150 mg/dL; high-density lipoprotein cholesterol <40 mg/dL in men or <50 mg/dL in women; and systolic/diastolic blood pressure ≥130/85 mmHg or ongoing antihypertensive treatment.

### Western Blot Analysis

Semiquantitative assessment of IAP expression in duodenal tissue was performed using immunoblotting techniques. Duodenal homogenates were separated by Sodium dodecyl sulfate–polyacrylamide gel electrophoresis and subsequently transferred onto polyvinylidene difluoride membranes. After blocking with 5% nonfat dry milk prepared in Tris-buffered saline containing 0.1% Tween-20, the membranes were incubated overnight at 4°C with a primary antibody against IAP (Abcam, UK; 1:1000 dilution). After incubation with a horseradish peroxidase–linked secondary antibody, immunoreactive bands were detected using an enhanced chemiluminescence system (Bio-Rad). Densitometric analysis was performed using ImageJ software, and protein expression levels were normalized to β-actin as an internal control.

### Statistical Analysis

Statistical analyses were performed using Python 3.13 (64-bit) (Python Software Foundation, Wilmington, DE, USA). Categorical variables were expressed as counts and percentages and compared using the Pearson chi-square test, with appropriate correction for multiple comparisons applied when necessary. Effect size for categorical variables was evaluated using Cramer’s V. Continuous variables were presented as median (interquartile range) because of distributional characteristics and sample size. Comparisons among the 3 groups were performed using the Kruskal–Wallis *H*-test. When significant differences were identified, post hoc pairwise comparisons with appropriate correction for multiple testing were conducted. Effect size was calculated using eta squared based on the Kruskal–Wallis statistic.

To visualize biomarker distributions, boxplots and jitter/strip plots were generated. Scatterplots with fitted linear regression lines were used to assess the associations between BMI and biomarker levels.

To evaluate the discriminative performance of biomarkers across BMI categories, multiclass receiver operating characteristic (ROC) analysis was performed using a one-vs-rest approach, adjusting for age and sex. ROC curves were generated from predicted probabilities derived from logistic regression models. The area under the curve (AUC) and 95% CIs were calculated using bootstrap resampling (2000 iterations), and statistical significance was assessed using permutation testing with 5000 iterations. Holm correction was applied for multiple comparisons. Optimal cut-off values were determined using the Youden index.

To obtain clinically interpretable thresholds, an additional conventional (unadjusted) ROC analysis was performed using raw biomarker values to compare obese and nonobese individuals.

In addition, multinomial logistic regression analysis was conducted to further support the ROC findings. The BMI category was included as the dependent variable, and biomarker levels were entered as independent variables adjusted for age and sex. The strength of associations was reported as odds ratios (ORs) with 95% CIs, allowing assessment of the independent effect of biomarkers on BMI category classification.

A *P*-value of <.05 was considered statistically significant. Written informed consent was obtained from all participants. The study was approved by the Ege University Clinical Research Ethics Committee on December 22, 2016 (Approval No. 16-12/6).

## Results

A total of 52 participants were included in the study and categorized as normal weight, overweight, and obese. Significant differences were observed among the groups in age (*P* = .034), height (*P* = .025), weight (*P* < .001), BMI (*P* < .001), and waist circumference (*P* < .001). Overweight individuals were older than normal-weight individuals. In contrast, no significant differences were observed in sex distribution, blood pressure, fasting plasma glucose, lipid parameters, or other clinical variables (all *P* > .05) (Table 1).

**Figure 2.**
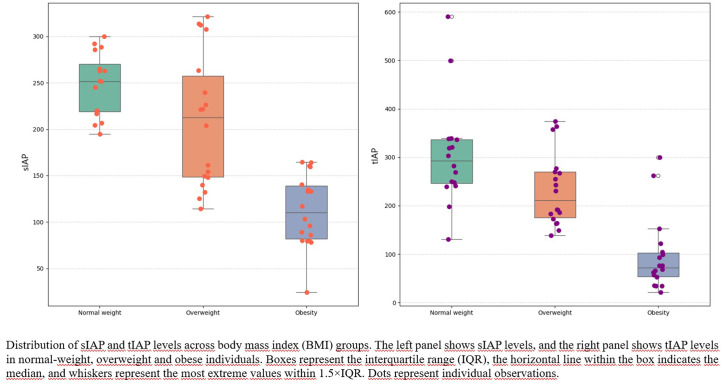
Distribution of intestinal alkaline phosphatase levels across body mass index groups.

Both sIAP and tIAP levels differed significantly among BMI groups (*P* < .001 for both) (Figure 2). Median sIAP levels were 251.58 ng/mL (218.96-270.17) in the normal-weight group, 212.66 ng/mL (148.64-257.46) in the overweight group, and 110.23 ng/mL (81.70-138.91) in the obese group. Similarly, tIAP levels were 292.97 ng/mL (246.29-336.76), 211.21 ng/mL (175.06-269.59), and 72.44 ng/mL, respectively. Post hoc analyses revealed that both biomarkers were significantly lower in obese individuals than in normal-weight and overweight groups (*P* < .05), whereas no significant difference was observed between normal-weight and overweight groups. Effect sizes were large (*η*^2^ = 0.521 for sIAP and *η*^2^ = 0.524 for tIAP), indicating strong associations between BMI category and biomarker levels (Table 2).

**Figure 3.**
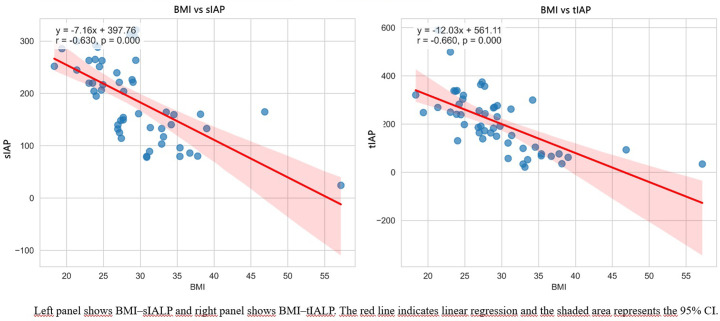
Association between body mass index and intestinal alkaline phosphatase levels.

BMI showed strong negative correlations with both biomarkers. Specifically, BMI was inversely correlated with sIAP (*r* = −0.630, *P* < .001) and tIAP (*r* = −0.660, *P* < .001), indicating that higher BMI values were associated with lower IAP levels (Figure 3).

Multinomial logistic regression analysis was performed to evaluate the independent association between biomarkers and BMI categories, with obesity used as the reference group.

In the model including tIAP, higher tIAP levels were independently associated with greater odds of belonging to the normal-weight group (OR = 1.037, 95% CI: 1.018-1.056, *P* < .001) and the overweight group (OR = 1.026, 95% CI: 1.009-1.043, *P* = .002). Age was not a significant predictor (*P* > .05). Female sex was associated with lower odds of belonging to the overweight group than to the obese group (OR = 0.023, 95% CI: 0.001-0.903, *P* = .044). The model was statistically significant (*χ*^2^(6) = 52.449, *P* < .001) and demonstrated high explanatory power (Nagelkerke *R*^2^ = 0.715) (Table 3). In the model including sIALP, similar findings were observed. Higher sIAP levels were associated with greater odds of belonging to the normal-weight group (OR = 1.069, 95% CI: 1.023-1.116, *P* = .003) and the overweight group (OR = 1.054, 95% CI: 1.011-1.098, *P* = .013). Female sex was associated with lower odds of belonging to both the normal-weight group (OR = 0.027, 95% CI: 0.001-0.978, *P* = .049) and the overweight group (OR = 0.023, 95% CI: 0.001-0.667, *P* = .028) than to the obese group. Age was not significantly associated with BMI category. This model also demonstrated strong fit (*χ*^2^(6) = 55.872, *P* < .001; Nagelkerke *R*^2^ = 0.741) (Table 4).

**Figure 4.**
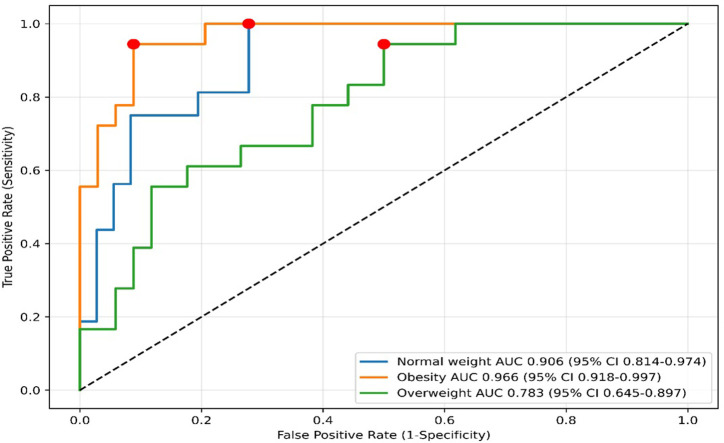
Age- and sex-adjusted receiver operating characteristic analysis of serum intestinal alkaline phosphatase for discrimination of body mass index groups. AUC, area under the curve.

**Figure 5.**
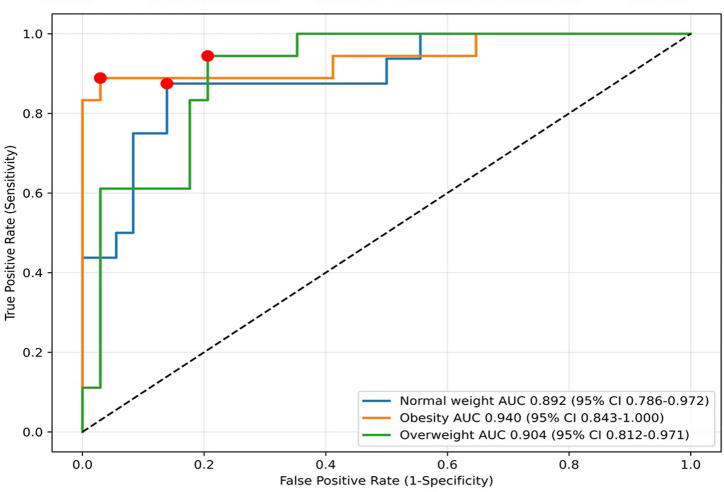
Age- and sex-adjusted receiver operating characteristic analysis of tissue intestinal alkaline phosphatase for discrimination of body mass index groups. AUC, area under the curve.

To complement and validate these regression-based findings from a discriminative perspective, ROC analysis was subsequently performed. This approach allowed evaluation of the ability of sIAP and tIAP to distinguish among BMI categories, thereby providing additional support for the classification performance suggested by the multinomial regression models. In age- and sex-adjusted multiclass ROC analysis, both biomarkers demonstrated high discriminative performance across BMI categories. For sIAP, AUC values were 0.906 ng/mL (95% CI: 0.814-0.974) for normal-weight individuals, 0.783 ng/mL (95% CI: 0.645-0.897) for overweight individuals, and 0.966 ng/mL (95% CI: 0.918-0.997) for obese individuals (Figure 4). For tIAP, AUC values were 0.892 ng/mL (95% CI: 0.786-0.972), 0.904 (95% CI: 0.812-0.971), and 0.940 ng/mL (95% CI: 0.843-1.000), respectively (Figure 5). All comparisons remained statistically significant after Holm correction (*P *≤ .001). Adjusted ROC-derived optimal cutoff probabilities for identifying obesity were 0.477 for sIALP and 0.487 for tIALP (Table 5).

In conventional (unadjusted) ROC analysis comparing obese and nonobese individuals, sIAP demonstrated an AUC of 0.928 with an optimal cutoff value of 164.81 ng/mL (sensitivity of 1.00 and specificity of 0.765). Similarly, tIAP showed an AUC of 0.928 with a cutoff value of 121.62 ng/mL (sensitivity of 0.833 and specificity of 1.00).

**Figure 6.**
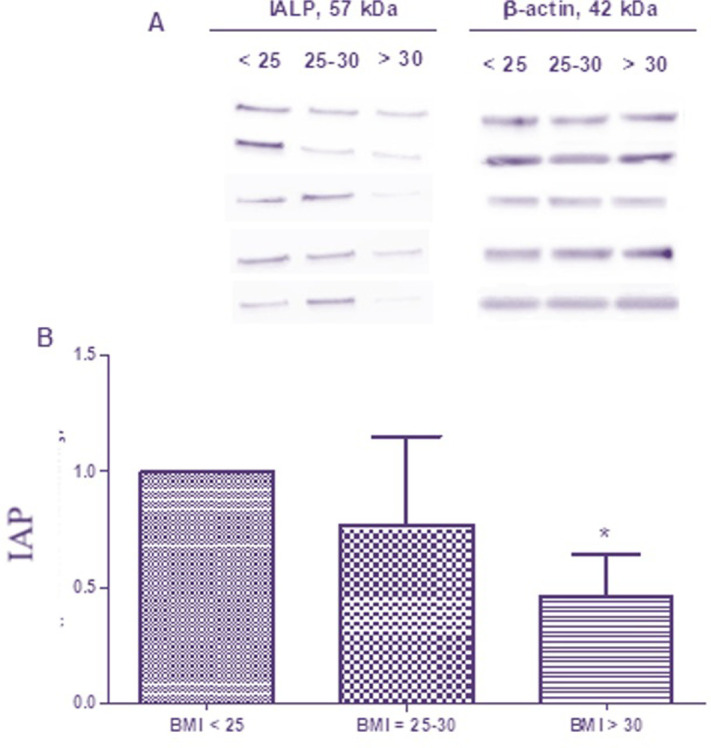
Western blot analysis of intestinal tissue intestinal alkaline phosphatase levels in individuals with different body mass index.

Following the qualitative analysis of enzyme levels, tIALP protein expression was evaluated using the Western blot method, which allows semiquantitative assessment. According to the findings, intestinal tIAP levels differed among BMI categories (Figure 6). No significant difference was observed between the normal-weight and the overweight groups (*P* > .05). On the other hand, IAP levels decreased with increasing BMI, and a significant reduction was observed in the obese group (*P* = .0139; BMI <25: 1.12 ± 0.11; BMI >30: 0.41 ± 0.18).

MeS was identified in 18 participants (34.6%). Subjects with MeS exhibited significantly lower tIAP levels (161.1 ± 106.5 ng/mL) than those without MeS (232.5 ± 124.9 ng/mL, *P* = .045). sIAP levels were also lower in the MeS group (170.2 ± 82.3 ng/mL vs. 197.9 ± 73.2 ng/mL), although this difference was not statistically significant (*P* = .093) (Table 6).

A total of 52 patients were analyzed according to ABO blood groups. Mean sIAP levels were 189.75 ± 88.34 ng/mL in group A (n = 19), 187.66 ± 84.00 ng/mL in group B (n = 14), 190.56 ± 45.24 ng/mL in group AB (n = 6), and 189.75 ± 88.34 ng/mL in group O (n = 13). No significant difference was found among groups (*P* > .05). Similarly, mean tIAP levels were 222.16 ± 131.30 ng/mL in group A, 185.61 ± 95.23 ng/mL in group B, 300.18 ± 161.42 ng/mL in group AB, and 167.98 ± 102.64 ng/mL in group O, with no statistically significant difference (*P* = .137). Smokers (n = 13) had a mean sIAP level of 217.51 ± 79.65 ng/mL, whereas nonsmokers (n = 39) had a mean sIAP level of 177.38 ± 74.78 ng/mL; however, this difference was not statistically significant (*P* = .102). Similarly, mean tIAP levels were 248.85 ± 86.72 ng/mL in smokers and 194.08 ± 130.59 ng/mL in nonsmokers, with no significant difference between the groups (*P* = .166). In terms of alcohol consumption, mean sIAP levels were 214.18 ± 55.68 ng/mL in alcohol users (n = 6) and 183.92 ± 78.64 ng/mL in nonusers (n = 46), without a statistically significant difference (*P* = .368). In contrast, mean tIAP levels are significantly higher in alcohol users (310.09 ± 65.54 ng/mL) than in nonusers (194.43 ± 122.48 ng/mL) (*P* = .028).

## Discussion

The present study comprehensively evaluated the association between sIAP and duodenal IAP levels and BMI in a well-characterized cohort. The findings demonstrated that IAP levels were associated with metabolic parameters across different BMI categories, supporting a potential link between IAP and overall metabolic status. Notably, this association was consistent when both circulating and tissue-level measurements were considered, suggesting that IAP may reflect both systemic and local intestinal processes. However, these findings should be interpreted with caution because the cross-sectional design of the study precludes any conclusions regarding causality or directionality. It remains unclear whether alterations in IAP levels contribute to the development of metabolic disturbances or represent a secondary response to underlying metabolic and inflammatory changes. Taken together, the results suggest that IAP may serve as a biologically relevant marker of metabolic and intestinal homeostasis rather than a direct indicator of adiposity, highlighting its potential role in the complex pathophysiology of obesity-related metabolic alterations.

IAP is a key brush-border enzyme involved in maintaining intestinal homeostasis through the detoxification of LPS and modulation of Toll-like receptor 4 (TLR4)-mediated inflammatory signaling.^11^By dephosphorylating LPS, IAP reduces endotoxin-induced activation of innate immune pathways and contributes to the preservation of intestinal barrier integrity. It has been demonstrated that IAP significantly affects intestinal permeability, as evidenced by its ability to reduce inflammation-induced barrier dysfunction and preserve tight junction protein expression and localization.^12^In this context, impaired IAP activity has been associated with increased intestinal permeability, low-grade systemic inflammation, and metabolic dysregulation.

Experimental studies have demonstrated that IAP plays a protective role against MeS, showing that both endogenous and exogenously administered IAP can inhibit intestinal endotoxin absorption and prevent as well as reverse high-fat diet–induced metabolic alterations.^13^Similarly, in models of liver disease, decreased IAP activity has been associated with impaired gut barrier function and progression of liver fibrosis, whereas IAP supplementation restores barrier integrity and attenuates fibrosis through a TLR4-dependent mechanism.^14^

In humans, reduced fecal IAP levels have been consistently associated with metabolic disorders. Early studies demonstrated an inverse relationship between fecal IAP levels and T2DM, with low IAP levels identified as an independent risk factor.^9^These findings were further supported by longitudinal data showing that persistent or incident IAP deficiency significantly increases the risk of developing T2DM over time, whereas higher IAP levels appear to be protective.^15^ In addition, lower fecal IAP levels have also been associated with an increased risk of ischemic heart disease, suggesting a broader role of IAP in cardiometabolic health.^16^

Beyond metabolic conditions, IAP has also been implicated in inflammatory and barrier-related disorders. Tissue-based human studies have demonstrated that duodenal IAP expression at both mRNA and protein levels is significantly reduced in newly diagnosed celiac disease and normalizes following a gluten-free diet.^8^ Similarly, decreased IAP expression in inflamed mucosa has been associated with worse clinical outcomes, including increased recurrence risk in Crohn’s disease.^17^ In line with these findings, reduced circulating IAP levels have been associated with increased markers of intestinal permeability and systemic inflammation, including lipopolysaccharide-binding protein, zonulin, and inflammatory indices such as the neutrophil-to-lymphocyte ratio.^12^

More recently, integrative human studies combining metabolic and intestinal parameters have shown that duodenal IAP activity is reduced in individuals with obesity and dysglycemia and is inversely correlated with glycemic markers such as fasting glucose and HbA1c.^13^ Supporting the role of IAP as a dynamic regulator, alterations in IAP activity have also been observed in inflammatory conditions such as severe coronavirus disease 2019, where changes in IAP kinetics may reflect a compensatory response to disrupted gut homeostasis.^18^Furthermore, experimental data indicate that IAP deficiency contributes to gut barrier dysfunction, microbial dysbiosis, and hepatic inflammation in the context of inflammatory bowel disease, promoting the development of non-alcoholic fatty liver disease via a TLR4-dependent mechanism.^19^

IAP plays a key role in regulating metabolic inflammation, and reduced activity has been associated with MeS in previous studies. In the current study, lower tIAP levels observed in patients with MeS are consistent with the existing literature. However, although sIAP levels were also lower, this difference did not reach statistical significance. The discrepancy between tIAP and sIAP levels may be explained by the fact that tIAP more directly reflects local intestinal production and mucosal barrier function, whereas circulating IAP represents a more heterogeneous and diluted pool influenced by systemic distribution, hepatic clearance, and other alkaline phosphatase isoenzymes. In MeS, alterations are likely to originate primarily at the level of the intestinal epithelium, leading to more pronounced and detectable changes in tissue measurements. In contrast, these local changes may not be fully reflected in the circulation, where the signal can be attenuated and masked by biological variability. Furthermore, the relatively small sample size may have limited the statistical power to detect modest differences in sIAP levels, even if a similar trend was present.

Collectively, these findings from experimental, clinical, and translational studies consistently support a biologically plausible link between IAP, intestinal barrier integrity, endotoxemia, and metabolic and systemic inflammatory processes, providing a comprehensive mechanistic framework for the associations observed in the study. Within this framework, the findings are consistent with the literature, suggesting that IAP is more closely related to metabolic and inflammatory alterations than to obesity per se. The heterogeneity of previous results may be partly explained by differences in measurement methods (fecal IAP, sIAP, or tIAP) and study populations. By evaluating both sIAP and tIAP levels, together with histochemical validation using Western blotting analysis, the study adds to the existing evidence of systemic and local IAP alterations in obesity.

A further important limitation of this study, in addition to its cross-sectional design and relatively small sample size, is the lack of detailed dietary assessment and preanalytical standardization. IAP levels are known to be influenced by multiple nutritional and physiological factors, including macronutrient composition, caloric intake, and feeding status, which may have introduced unmeasured variability into the findings. Experimental and translational evidence indicates that nutrients such as dietary fats, carbohydrates, amino acids, vitamins, and probiotics can significantly modulate IAP expression and activity as well as intestinal barrier function.^20^In addition, IAP levels may vary between fasting and postprandial states, reflecting dynamic regulation in response to luminal stimuli and endotoxin exposure.^21^ Therefore, the lack of standardized dietary conditions and timing of sample collection may have introduced variability in IAP measurements and may have influenced the observed associations. Additionally, although alcohol consumption^22^ and smoking are known to influence IAP levels, and despite the low number of individuals with alcohol use and smoking in this cohort, the findings did not demonstrate a significant association between smoking status and either sIAP or tIAP levels. Similarly, alcohol consumption was not associated with sIAP levels; however, tIAP levels were significantly higher in individuals who consume alcohol. Notably, both smoking and alcohol users tended to exhibit numerically higher IAP levels overall, although this does not reach statistical significance for smoking. One possible explanation is that IAP expression may increase as a compensatory response to luminal inflammatory stimuli. In particular, acute alcohol exposure can disrupt intestinal barrier integrity and increase endotoxin translocation, potentially triggering a protective upregulation of IAP. Thus, the elevated tIAP levels observed in alcohol users may reflect a reactive response to increased luminal inflammation rather than a direct causal effect. These findings should be interpreted with caution, as the relatively low prevalence of smoking and alcohol use in the cohort and the cross-sectional design limit the robustness and causal interpretation of these associations. Therefore, larger studies with balanced cohorts, controlled dietary conditions, and standardized sampling protocols are warranted to better clarify the relationship between IAP, lifestyle factors, and metabolic parameters.

Future studies should aim to clarify the causal relationship between IAP and metabolic disorders through well-designed prospective and longitudinal investigations. Standardization of preanalytical conditions, including dietary intake and sampling timing, will be essential to reduce variability and better characterize the biological role of IAP. In addition, integrating multi-compartment measurements such as sIAP, tIAP, and fecal IAP, may provide a more comprehensive understanding of IAP dynamics and its compartment-specific functions. Moreover, given emerging evidence suggesting that IAP activity can be modulated by nutritional and pharmacological interventions, future research should explore whether targeted modulation of IAP—either through dietary strategies or exogenous supplementation—could have therapeutic potential in metabolic diseases.

In conclusion, the findings demonstrate that IAP is associated with metabolic parameters in humans and may reflect underlying intestinal barrier dysfunction and metabolic inflammation. IAP appears to be linked to the broader metabolic milieu, including glucose metabolism and low-grade inflammatory processes. These results are consistent with accumulating evidence suggesting a role for IAP in maintaining gut homeostasis and modulating endotoxin-mediated pathways. However, given the observational design of the study, these associations should be interpreted with caution. Future prospective and mechanistic studies are needed to clarify the causal role of IAP and to determine whether modulation of IAP activity could represent a potential therapeutic strategy in metabolic disorders.

## Figures and Tables

**Table 1.** Comparison of Demographic and Clinical Characteristics Between Groups

	Normal Weight	Overweight	Obesity	*η*^2^/Cramer’s *V*	
Age	51.50 (31.00-58.25)	62.50 (53.50-70.50)	53.50 (42.50-59.00)	.034	0.097^a^
Sex (female/male)	10 (62.5)/6 (37.5)	9 (50.0)/9 (50.0)	15 (83.3)/3 (16.7)	.105	0.294^b^
Height (m)	1.70 (1.66-1.73)	1.67 (1.60-1.70)	1.60 (1.50-1.67)	.025	0.110^c^
Weight (kg)	67.50 (59.00-71.00)	79.50 (71.50-84.00)	84.00 (79.25-107.00)	<.001	0.377^d^
BMI (kg/m^2^)	23.80 (22.63-24.28)	27.71 (27.21-28.98)	34.37 (32.89-37.51)	<.001	0.884^e^
WC (cm)	85.50 (82.25-98.00)	102.50 (100.25-108.50)	106.00 (101.00-116.00)	<.001	0.430^d^
sBP (mmHg)	115.00 (109.50-121.50)	135.00 (110.00-140.75)	127.00 (121.25-136.50)	.059	0.074
dBP (mmHg)	74.50 (68.00-82.50)	80.00 (69.25-91.00)	78.50 (71.50-83.25)	.618	−0.021
FBG (mg/dL)	89.00 (85.00-93.25)	88.50 (85.25-95.75)	89.50 (81.25-95.75)	.956	−0.039
HDL (mg/dL)	48.00 (34.00-55.50)	46.00 (40.25-57.00)	47.00 (40.00-56.00)	.866	−0.035
TGL (mg/dL)	112.00 (81.25-135.25)	111.50 (81.00-151.25)	168.50 (110.25-202.00)	.197	0.025
ALP (U/dL)	69.00 (60.25-72.50)	74.00 (61.50-86.50)	72.50 (66.00-91.50)	.257	0.015
Smoker	6 (37.5)	4 (22.2)	3 (16.7)	.355	0.200
Alcohol user	3 (18.8)	3 (16.7)	0 (0.0)	.163	0.264

Categorical variables are presented as n (%) and compared using the Pearson chi-square test, with Cramer's *V* reported as the effect size. Continuous variables are presented as median (interquartile range) and compared across the 3 groups using the Kruskal–Wallis *H*-test. Effect size for the Kruskal–Wallis test was calculated using eta squared (*η*^2^ = (*H* − *k* + 1)/(*N* − *k*)). When a significant overall difference was detected, Dunn’s post hoc test with Bonferroni correction was applied for pairwise comparisons. All *P* values are 2-sided and reported to 3 decimal places.

ALP, alkaline phosphatase; BMI, body mass index; dBP, diastolic blood pressure; FBG, fasting blood glucose; HDL, high-density lipoprotein; sBP, systolic blood pressure; TGL, triglycerides; WC, waist circumference.

^a^Overweight > Normal weight.

^b^Obesity > Overweight.

^c^Obesity < Overweight.

^d^Obesity > Overweight, Normal weight.

^e^Obesity > Overweight > Normal weight.

**Table 2.** Comparison of sIAP and tIAP Levels Among BMI Groups

	Normal Weight	Overweight	Obesity	*P*	*η* ^2^	Post Hoc
sIAP (ng/mL)	251.58 (218.96-270.17)	212.66 (148.64-257.46)	110.23 (81.70-138.91)	<.001	0.521	1-3, 2-3
tIAP (ng/mL)	292.97 (246.29-336.76)	211.21 (175.06-269.59)	72.44 (53.65-103.02)	<.001	0.524	1-3, 2-3

Continuous variables are presented as median (interquartile range) and compared across the 3 groups using the Kruskal–Wallis *H*-test. Effect size for the Kruskal–Wallis test was calculated using eta squared (*η*^2^ = (*H* − *k* + 1)/(*N* − *k*)). When a significant overall difference was detected, Dunn’s post hoc test with Bonferroni correction was applied for pairwise comparisons. All *P* values are 2-sided and reported to 3 decimal places.

BMI, body mass index; sIAP, serum intestinal alkaline phosphatase; tIAP, tissue intestinal alkaline phosphatase.

**Table 3.** Multinomial Logistic Regression Model

Variable	Comparison	OR	95% GA	*P*
Age	1 vs. 3	1.036	0.928-1.157	.529
	2 vs. 3	1.106	0.989-1.238	.078
tIAP	1 vs. 3	**1.037**	1.018-1.056	**<.001**
	2 vs. 3	**1.026**	1.009-1.043	**.002**
Sex (female)	1 vs. 3	0.077	0.002-3.303	.181
	2 vs. 3	**0.023**	0.001-0.903	**.044**

Model fit statistics: Likelihood ratio *χ*^2^(6) = 52.449; *P* < .001. Pseudo *R*^2^: Cox ve Snell *R*^2^ = 0.635; Nagelkerke *R*^2^ = 0.715; McFadden *R*^2^ = 0.460. 1, normal-weight group; 2, overweight group; 3, obese group. Bold values indicate statistically significant results (p < 0.05).

GA, generalized additive; OR, odds ratio; tIAP, tissue intestinal alkaline phosphatase.

**Table 4.** Multinomial Logistic Regression Model

Variable	Comparison	OR	95% GA	*P*
Age	1 vs. 3	1.012	0.906-1.131	.830
	2 vs. 3	1.079	0.967-1.204	.176
sIAP	1 vs. 3	**1.069**	1.023-1.116	**.003**
	2 vs. 3	**1.054**	1.011-1.098	**.013**
Sex (female)	1 vs. 3	**0.027**	0.001-0.978	**.049**
	2 vs. 3	**0.023**	0.001-0.667	**.028**

Model fit statistics: Likelihood ratio *χ*^2^(6) = 55.872; *P* < .001. Pseudo *R*^2^: Cox ve Snell *R*^2^ = 0.659; Nagelkerke *R*^2^ = 0.741; McFadden *R*^2^ = 0.490. 1, normal-weight group, 2, overweight group; 3, obese group.

.

GA, generalized additive; OR, odds ratio; sIAP, serum intestinal alkaline phosphatase.

**Table 5.** ROC Analysis Results (Adjusted for Age and Sex)

	Group	AUC	95% GA	*P* (Permutation)	*P* (Holm Adjusted)	Cutoff Value (Adjusted ROC)
sIAP	Normal weight	0.906	0.814-0.974	<.001	.001	0.192
	Obesity	0.966	0.918-0.997	<.001	<.001	0.477
	Overweight	0.783	0.645-0.897	.001	.001	0.225
tIAP	Normal weight	0.892	0.786-0.972	<.001	.001	0.399
	Obesity	0.940	0.843-1.000	<.001	<.001	0.487
	Overweight	0.904	0.812-0.971	<.001	<.001	0.383

AUC, area under the curve; GA, generalized additive; ROC, receiver operating characteristic; sIAP, serum intestinal alkaline phosphatase; tIAP, tissue intestinal alkaline phosphatase.

**Table 6.** sIAP and tIAP Levels in Subjects with and Without Metabolic Syndrome

Variable	MeS (+) (n = 18)	MeS (−) (n = 34)	*P*
sIAP (ng/mL)	170.2 ± 82.3	197.9 ± 73.2	.093
tIAP (ng/mL)	161.1 ± 106.5	232.5 ± 124.9	.045

IAP, intestinal alkaline phosphatase; MeS, metabolic syndrome; mL, milliliter; ng, nanograms; sIAP, serum IAP; tIAP, tissue IAP.

## Data Availability

The data that support the findings of this study are available on request from the corresponding author.

## References

[1] Suzuki U Yoshimura K Takaishi M. Ueber ein Enzym "Phytase", das "anhydro-oxy-methylen Diphosphorsäure" spaltet about the enzyme “phytase”, which splits “anhydro-oxy-methylene diphosphoric acid”. Noka-Daigaku-Gakujutsu-Hokoku. Tokyo Imperial University. 2008;7:503 512.

[2] Sharma U Pal D Prasad R. Alkaline phosphatase: an overview. Indian J Clin Biochem. 2014;29(3):269 278. (doi: 10.1007/s12291-013-0408-y) 24966474 PMC4062654

[3] Lallès JP. Intestinal alkaline phosphatase: novel functions and protective effects. Nutr Rev. 2014;72(2):82 94. (doi: 10.1111/nure.12082) 24506153

[4] Lallès JP. Intestinal alkaline phosphatase: multiple biological roles in maintenance of intestinal homeostasis and modulation by diet. Nutr Rev. 2010;68(6):323 332. (doi: 10.1111/j.1753-4887.2010.00292.x) 20536777

[5] Narisawa S Huang L Iwasaki A Accelerated fat absorption in intestinal alkaline phosphatase knockout mice. Mol Cell Biol. 2003;23(21):7525 7530. (doi: 10.1128/MCB.23.21.7525-7530.2003) 14560000 PMC207564

[6] Nakano T Inoue I Koyama I Disruption of the murine intestinal alkaline phosphatase gene Akp3 impairs lipid transcytosis and induces visceral fat accumulation and hepatic steatosis. Am J Physiol Gastrointest Liver Physiol. 2007;292(5):G1439 G1449. (doi: 10.1152/ajpgi.00331.2006) 17332477

[7] Kaliannan K Hamarneh SR Economopoulos KP Intestinal alkaline phosphatase prevents metabolic syndrome in mice. Proc Natl Acad Sci U S A. 2013;110(17):7003 7008. (doi: 10.1073/pnas.1220180110) 23569246 PMC3637741

[8] Molnár K Vannay A Sziksz E Decreased mucosal expression of intestinal alkaline phosphatase in children with coeliac disease. Virchows Arch. 2012;460(2):157 161. (doi: 10.1007/s00428-011-1188-5) 22262031

[9] Malo MS. A high level of intestinal alkaline phosphatase is protective against type 2 diabetes mellitus irrespective of obesity. EBiomedicine. 2015;2(12):2016 2023. (doi: 10.1016/j.ebiom.2015.11.027) 26844282 PMC4703762

[10] Fawley J Gourlay DM. Intestinal alkaline phosphatase: a summary of its role in clinical disease. J Surg Res. 2016;202(1):225 234. (doi: 10.1016/j.jss.2015.12.008) 27083970 PMC4834149

[11] Bates JM Akerlund J Mittge E Intestinal alkaline phosphatase detoxifies lipopolysaccharide and prevents inflammation in zebrafish in response to the gut microbiota. Cell Host Microbe. 2007;2(6):371 382. (doi: 10.1016/j.chom.2007.10.010) 18078689 PMC2730374

[12] Liu W Hu D Huo H Intestinal alkaline phosphatase regulates tight junction protein levels. J Am Coll Surg. 2016;222(6):1009 1017. (doi: 10.1016/j.jamcollsurg.2015.12.006) 27106638 PMC5684582

[13] Lopes KG de Souza MD Marques Lopes FA Gut microbiota, intestinal barrier function, and metabolism across adiposity and glucose tolerance. Nutrients. 2025;17(21):3380. (doi: 10.3390/nu17213380) PMC12610977 41228453

[14] Liu Y Cavallaro PM Kim BM A role for intestinal alkaline phosphatase in preventing liver fibrosis. Theranostics. 2021;11(1):14 26. (doi: 10.7150/thno.48468) 33391458 PMC7681079

[15] Malo J Alam MJ Islam S Intestinal alkaline phosphatase deficiency increases the risk of diabetes. BMJ Open Diabetes Res Care. 2022;10(1):e002643. (doi: 10.1136/bmjdrc-2021-002643) PMC8796214 35082135

[16] Malo J Alam MJ Shahnaz M Intestinal alkaline phosphatase deficiency is associated with ischemic heart disease. Dis Markers. 2019;2019:8473565. (doi: 10.1155/2019/8473565) PMC6930721 31915470

[17] Park SY Kim JY Lee SM Lower expression of endogenous intestinal alkaline phosphatase may predict worse prognosis in patients with Crohn's disease. BMC Gastroenterol. 2018;18(1):188. (doi: 10.1186/s12876-018-0904-x) PMC6296121 30558547

[18] Araújo JR Serafim T Ismael S Intestinal alkaline phosphatase activity and efficiency are altered in severe COVID-19 patients. Gastro Hep Adv. 2023;2(7):911 917. (doi: 10.1016/j.gastha.2023.07.003) 39130768 PMC11307804

[19] Liu Y Lv H Zhang H The protective role of intestinal alkaline phosphatase in inflammatory bowel disease-associated non-alcoholic fatty liver disease. Life Sci. 2026;384:124113. (doi: 10.1016/j.lfs.2025.124113) 41297668

[20] Alvarenga L Cardozo LFMF Lindholm B Intestinal alkaline phosphatase modulation by food components: predictive, preventive, and personalized strategies for novel treatment options in chronic kidney disease. EPMA J. 2020;11(4):565 579. (doi: 10.1007/s13167-020-00228-9) 33240450 PMC7680467

[21] Gao C Koko MY Hong W Protective properties of intestinal alkaline phosphatase supplementation on the intestinal barrier: interactions and effects. J Agric Food Chem. 2024;72(1):27 45. (doi: 10.1021/acs.jafc.3c05119) 37964463

[22] Hamarneh SR Kim BM Kaliannan K Intestinal alkaline phosphatase attenuates alcohol-induced hepatosteatosis in mice. Dig Dis Sci. 2017;62(8):2021 2034. (doi: 10.1007/s10620-017-4576-0) 28424943 PMC5684583

